# Individualized Endurance Training Based on Recovery and Training Status in Recreational Runners

**DOI:** 10.1249/MSS.0000000000002968

**Published:** 2022-08-17

**Authors:** OLLI-PEKKA NUUTTILA, ARI NUMMELA, ELISA KORHONEN, KEIJO HÄKKINEN, HEIKKI KYRÖLÄINEN

**Affiliations:** 1 Faculty of Sport and Health Sciences, University of Jyväskylä, Jyväskylä, FINLAND; 2 Finnish Institute of High Performance Sport KIHU, Jyväskylä, FINLAND

**Keywords:** ENDURANCE PERFORMANCE, RUNNING PERFORMANCE, HEART RATE VARIABILITY, PERCEIVED RECOVERY, PERIODIZATION

## Abstract

**Purpose:**

Long-term development of endurance performance requires a proper balance between strain and recovery. Because responses and adaptations to training are highly individual, this study examined whether individually adjusted endurance training based on recovery and training status would lead to greater adaptations compared with a predefined program.

**Methods:**

Recreational runners were divided into predefined (PD; *n* = 14) or individualized (IND; *n* = 16) training groups. In IND, the training load was decreased, maintained, or increased twice a week based on nocturnal heart rate variability, perceived recovery, and heart rate–running speed index. Both groups performed 3-wk preparatory, 6-wk volume, and 6-wk interval periods. Incremental treadmill tests and 10-km running tests were performed before the preparatory period (*T*_0_) and after the preparatory (*T*_1_), volume (*T*_2_), and interval (*T*_3_) periods. The magnitude of training adaptations was defined based on the coefficient of variation between *T*_0_ and *T*_1_ tests (high >2×, low <0.5×).

**Results:**

Both groups improved (*P* < 0.01) their maximal treadmill speed and 10-km time from *T*_1_ to *T*_3_. The change in the 10-km time was greater in IND compared with PD (−6.2% ± 2.8% vs −2.9% ± 2.4%, *P* = 0.002). In addition, IND had more high responders (50% vs 29%) and fewer low responders (0% vs 21%) compared with PD in the change of maximal treadmill speed and 10-km performance (81% vs 23% and 13% vs 23%), respectively.

**Conclusions:**

PD and IND induced positive training adaptations, but the individualized training seemed more beneficial in endurance performance. Moreover, IND increased the likelihood of high response and decreased the occurrence of low response to endurance training.

Successful endurance training requires a proper balance between training load and recovery. Although adequate training stimulus is necessary to induce favorable adaptations, inadequate recovery between training sessions and periods may lead to excessive fatigue, and if the imbalance is extended, even to nonfunctional overreaching or overtraining (1). It has been observed that acute responses and recovery kinetics to similar training sessions (2–4) as well as adaptations to training periods (5–7) vary between individuals, and processes related to adaptation could be affected by multiple factors not connected with actual training, such as nutrition (8), sleep (9), or psychological stress (10). Therefore, monitoring both training and recovery could help to take individual differences into account and in this way provide useful information for the estimation of proper training load in each case (11).

The evolution of wearable technology has produced more options for the monitoring of training and recovery, which in turn makes individual training approaches more feasible. Lately, heart rate variability (HRV)–guided training has been utilized in various populations, leading to more beneficial training effects compared with predefined training in untrained (12), recreationally trained (13–15), and well-trained (16,17) participants. The assumption in HRV is that because it reflects the cardiac parasympathetic nervous system activity, it would also relate to current readiness to adapt to training stimulus (18). The basic idea in all studies utilizing the HRV-guided approach has been similar—training intensity has been modified based on changes in daily recorded resting HRV with respect to the individually defined reference range. Furthermore, values below and above the normal range have been regarded as a sign of an abnormal state, and only low-intensity training has been prescribed until HRV has reached the individual reference value (14,16,17). Interestingly, none of the previous studies have tried to manipulate training volume based on HRV, although it is an important variable in the endurance training prescription (19).

Despite the fact that HRV-guided training has induced some promising results, a single marker could not establish all aspects critical to recovery. Although HRV mainly reflects cardiac autonomic nervous system activity and cardiovascular homeostasis, aspects such as muscle tissue repair or muscle glycogen repletion may not necessarily be aligned with the parasympathetic reactivation (18). Indeed, neuromuscular and perceptual recovery has differed from the pattern of HRV in several studies (3,4,18,20). It can also be argued that training adaptation and HRV (or its responses) may not be as directly associated as sometimes it has been assumed (21), especially, when taking into consideration the challenging interpretation of HRV after intensified training (22,23) and the possible influence of plasma volume expansion (18). Therefore, supplementary monitoring methods providing information on perceived fatigue and musculoskeletal strain could help to gain a more comprehensive picture of the recovery status. To the best of our knowledge, only one previous study has considered multiple variables in the training decision scheme by analyzing the rating of perceived exertion, the ability to reach target heart rate (HR), and the HR recovery from a submaximal cycling test (24). Although it seems obvious that combining both objective and subjective markers would provide the best quality for monitoring, there certainly exists a lack of research on how to implement such an approach in practice.

To investigate the effectiveness of individualized training volume and intensity, the present study compared the individually adjusted training prescription based on nocturnal HRV, perceived recovery, and estimated running performance to the predefined training program in recreationally endurance-trained males and females. We hypothesized that individualized training would induce greater training adaptations in maximal running performance compared with predefined training and decrease the likelihood of low response.

## METHODS

### Participants

A total of 40 recreationally endurance-trained males (20) and females (20) were recruited for the study. The minimal sample size was determined based on the data of Nuuttila et al. (15) where 5.1% ± 3.2% and 2.7% ± 1.6% changes in maximal treadmill velocity were reported in HRV-guided and predefined groups, respectively. *A priori* power analysis suggested that 15 subjects were required for both groups to achieve 80% power and a significance level of 5%. The participants were healthy and accustomed to regular running (at least 4 times a week). Before the final acceptance to participate, a cardiologist checked the electrocardiography of all participants. During the study period, seven dropouts occurred (three during volume period, four during interval period). Dropouts in the predefined (PD) and individualized (IND) groups occurred because of personal reasons (*n* = 1/IND), illnesses (*n* = 2/PD, 1/IND), or injuries of the lower extremities (*n* = 2/PD, 1/IND). In addition, three participants that finished the study were excluded from the final analysis because of insufficient training adherence (<90% of the main sessions, *n* = 1/PD), prolonged training interruption during the interval period (>2 wk, *n* = 1/IND), or prolonged illness between the end of the interval period and the last testing week (>2 wk, *n* = 1/PD). The baseline characteristics of the participants that were included in the final analysis are presented in Table 1. One participant got sick between the last incremental treadmill test and the 10-km running test, and that is why in 10-km performance, *n* = 13 in PD. All participants gave their written consent to participate, and the study protocol was approved by the ethics committee of the University of Jyväskylä.

**TABLE 1:** Mean ± SD baseline characteristics of the participants.

	PD			IND		
	Males (*n* = 7)	Females (*n* = 7)	All (*n* = 14)	Males (*n* = 8)	Females (*n* = 8)	All (*n* = 16)
Age (yr)	33 ± 6	35 ± 8	34 ± 7	37 ± 5	38 ± 9	37 ± 7
Height (cm)	181 ± 3	168 ± 5	174 ± 8	180 ± 5	167 ± 8	174 ± 9
Body mass (kg)	82 ± 11	64 ± 8	73 ± 13	77 ± 12	59 ± 4	68 ± 13
BMI (kg·m^−2^)	25.0 ± 3.5	22.7 ± 3.3	23.9 ± 3.5	23.7 ± 3.7	21.3 ± 1.2	22.5 ± 3.0
Fat (%)	14.9 ± 5.1	21.0 ± 7.1	17.9 ± 6.7	12.0 ± 5.6	19.8 ± 4.8	15.9 ± 6.5
V̇O_2max_ (mL·kg^1^·min^−1^)	47.9 ± 4.2	43.4 ± 2.5	45.7 ± 4.0	50.6 ± 6.2	42.3 ± 4.3	46.5 ± 6.7

Baseline characteristics were measured before the preparatory period (*T*_0_).

BMI, body mass index.

### Study protocol

The study period consisted of a 3-wk preparatory period (PREP), which was followed by 6-wk volume (VOL) and interval periods (INT). After PREP, the participants were matched into pairs based on sex, endurance performance (maximal treadmill speed, 10 km), and endurance training volume (*h*); and after that, they were randomized into a PD group and an IND group. PD trained according to the predefined program, whereas the program of IND was adapted based on measured training and recovery data. In both groups, all the programmed sessions were performed by running. The participants were allowed to continue other regular activities (e.g., cycling, muscular fitness) with a similar proportion they were accustomed to. However, only a marginal number of such sessions were reported during the study (0.2 ± 0.3 sessions per week). Laboratory measurements and endurance performance tests were performed four times during a testing week before PREP (*T*_0_), between PREP and VOL (*T*_1_), between VOL and INT (*T*_2_), and after INT (*T*_3_). In addition, all participants collected HR and Global Positioning System data from endurance exercises, recorded daily their nocturnal HR and HRV, and filled questionnaires on perceived recovery. Training was performed in field conditions and mainly outdoors. Data collection was executed between late spring and autumn to ensure the most suitable conditions for running. The average daily peak temperature during the data collection was 17.3°C ± 7.5°C in the local weather station (FMI catalog, assessed 12.5.2022). Individuals were not given any specific guidelines regarding nutrition or fluid intake during the study period, and the aim was to maintain usual nutritional habits.

### Training protocol

During the 3-wk PREP, participants were familiarized with the intensity zones and training modes of the following periods. The PREP period also facilitated the assessment of the regular training volume of the participants and the representative individual baseline for the measured recovery variables. The participants were advised to continue their regular training in terms of volume and frequency. However, they were asked to exercise only at low intensity (LIT) except for one weekly predefined moderate-intensity session (MOD). To ensure sufficient recovery before the testing week that preceded the training intervention (*T*_1_), the participants were asked to decrease training volume by 25% during their last week of PREP. The training volume and training frequency were analyzed from this period for each individual and used as a basis in the following training programs.

After PREP, the PD and IND groups trained according to their programs. The first 6-wk VOL period focused on the progression of LIT volume, whereas the second 6-wk INT period focused on high-intensity interval training (HIT). The training program of PD was individually scaled based on the training frequency and volume during PREP. The basic structure of the program is presented in Table 2. The training modes during VOL included LIT sessions where HR was below the first lactate threshold (HRzone1) and continuous MOD sessions where HR was between the first and second lactate thresholds (HRzone2). The training was periodized in a way that 2 intensive weeks were followed by 1 recovery week. The training volume progression was similar to previous studies (7,25): during intensive weeks, it increased by 10% compared with the baseline level (2 first weeks of PREP). To ensure sufficient recovery, training volume was always decreased by 25% after 2 intensive weeks (26).

**TABLE 2:** The training program of the PD group during the preparatory (PREP1–3), volume (VOL1–6), and interval (INT1–6) training periods.

Week	LIT (Basic), 30–90 min	LIT (Long), >90 min	MOD, 30 min	HIT, 6 × 3 min	Tests V̇O_2max_, 10 km	Volume
*T*_0_	1–3×				x	
PREP1	2–4×	1×	1×			BL
PREP2	2–4×	1×	1×			BL
PREP3	1–3×	1×	1×			0.75 × BL
*T*_1_	1–3×				×	
VOL1	2–4×	1×	1×			1.1 × BL
VOL2	2–4×	1×	1×			1.2 × BL
VOL3	1–3×	1×	1×			0.75 × previous wk
VOL4	2–4×	1×	1×			1.3 × BL
VOL5	2–4×	1×	1×			1.4 × BL
VOL6	1–3×	1×	1×			0.75 × previous wk
*T*_2_	1–3×				×	
INT1	1–3×			3×		BL (basic sessions)
INT2	1–3×			3×		BL (basic sessions)
INT3	2–4×			1×		0.75 × previous wk
INT4	1–3×			3×		BL (basic sessions)
INT5	1–3×			3×		BL (basic sessions)
INT6	2–4×			1×		0.75 × previous wk
*T*_3_	1–3×				×	

All MOD and HIT sessions also included low-intensity warm-up and cool-down.

BL, baseline; LIT, low-intensity training (HRzone1); MOD, moderate-intensity training (HRzone2); HIT, high-intensity interval training (maximal sustainable effort); V̇O_2max_, incremental treadmill test.

During INT, the weekly main session was 6 × 3 min performed at the maximal sustainable effort with 2-min recovery intervals in between (27). Basically, the running speed during the intervals was between the second lactate threshold and maximal treadmill test speed and at the end of intervals, HR reached values above the second lactate threshold (HRzone3). Other endurance training was executed as LIT where HR was below the first lactate threshold (HRzone1). The duration of these sessions was individually defined based on the basic sessions` average values during PREP. Similar to VOL, the training was periodized into 2 intensive weeks (three HIT sessions) followed by 1 recovery week (one HIT session and 25% decreased training volume). The weekly HIT frequency was based on previous studies using 2–3 weekly HIT sessions (28,29).

**FIGURE 1:**
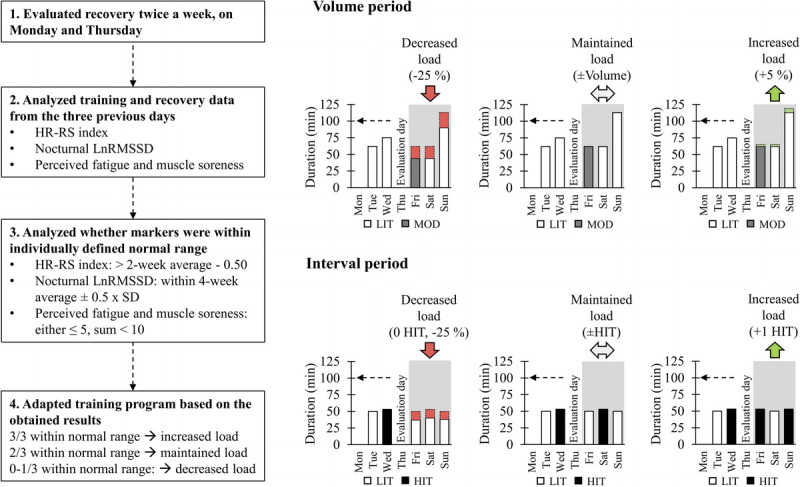
Determination logic of the training load in the IND group. Training load was adjusted twice a week on evaluation days (Monday and Thursday). If the training load was maintained, no modifications were made compared with the current level. The training load was increased via adding volume (VOL) by 5% (e.g., 1.10 × baseline level to 1.15 × baseline level) or via increasing the number of HIT sessions (INT). The training load was decreased via reducing volume by 25% compared with the current level (VOL), or via reducing volume by 25% from the current level and excluding HIT sessions (INT). After the recovery block, the training continued from the level preceding the recovery block (two-thirds of the markers within normal range) or the next level (VOL). During INT, the progression always started from one HIT. After reaching a maximum number of HIT sessions within a block (two or three sessions), no additional sessions were performed. After the last evaluation day of INT, a maximum of one HIT session was performed to ensure sufficient recovery before final tests.

In the IND group, the training frequency and timing of different types of sessions within a week were determined according to similar principles as in the PD group. Only the duration of the sessions (VOL) or the number of HIT sessions (INT) were adjusted based on the training and recovery state. The execution of the training was individually adjusted twice a week on evaluation days (Monday and Thursday), which were always recovery days (rest or active recovery) as well. Basically, the training load of the following 3- to 4-d block was either increased, maintained, or decreased from the current level set for the individual. During VOL, the current level referred to the coefficient of the session duration compared with baseline, and similar to PD, it started from +10% (1.10 × baseline duration). During INT, the current level referred to the number of HIT sessions performed within a block and started from one HIT, like in PD. The adjustment logic for the training load is illustrated in Figure 1. The participants were not informed about the exact model behind the training modification to avoid manipulation of the results in a way that would not be related to the actual recovery and training state.

The variables affecting the training load and their desirable ranges were determined in conformity with previous studies. In the nocturnal HRV, a 4-wk rolling average ±0.5 × SD was chosen, which meant that the values above or below the range were regarded as negative. Similar cutoff values have been used in studies utilizing HRV-guided training (14,16). Fatigue was expected to be sensitive for the (too high) changes in the training load (22,23) and to increase as a sign of possible overreaching (30,31). Muscle soreness has also increased after periods of intensified training (23,27,32), and high values may relate to overtraining (30). Hooper et al. (30) suggested that in a 1–7 scale, values >5 would be associated with staleness. Because the present study used a similar scale, the respective value was chosen as a cutoff for “normal” value. HR–running speed index (HR–RS index) was chosen as the third factor affecting the training load, because it is not straightforward how recovery state itself translates into training adaptation, and changes in this marker have previously correlated with the change in maximal running performance measured in the laboratory (33). Because HR–RS index was not measured in laboratory conditions and exercise HR has a certain natural day-to-day variation (34), the maximum decrement of 0.50 compared with previous 2-wk average, equivalent to 3- to 4-bpm increase in HR at the same running speed, was defined as “normal.” The smallest worthwhile change (SWC) of 0.50 has also been used with the same marker previously (21).

### Performance and laboratory tests

The testing week included 2 testing days, which were separated by at least 48 h. The first testing day consisted of fasting measurements (blood samples and anthropometrics) and incremental treadmill test. On the second day, a 10-km running test was executed. The tests were performed at the same time of the day (±2 h) within-participant. The last day before the test was a rest day and no HIT or long-distance sessions were performed on 2 d preceding any test.

Serum free testosterone and cortisol concentrations as well as creatine kinase activity were assessed after a 12-h fast in the morning (7:15–9:15 am) preceding the incremental treadmill test. Samples were taken from the antecubital vein into 6-mL serum tubes, and standard laboratory procedures were followed. Whole blood was centrifuged at 2250 G (Megafuge 1.0 R; Heraeus, Hanau, Germany) for 10 min, and after that the serum was removed and frozen at −20°C until the final analysis. Serum cortisol concentration was analyzed with a chemical luminescence technique (Immulite 2000 XPi; Siemens, New York City, NY). The sensitivity of the cortisol assay was 5.5 nmol·L^−1^, and the intra-assay coefficient of variation (CV) was 5.3%. Free testosterone concentration was analyzed with enzyme-linked immunoassay method (DYNEX DS 2 ELISA processing system; DYNEX Technologies, Chantilly, VA). The sensitivity of the free testosterone assay was 0.6 pmol·L^−1^, and the intra-assay CV was 6.0%. Serum creatine kinase activity was analyzed with Indiko Plus Clinical Chemistry Analyzer (Thermo Fisher Scientific, Vantaa, Finland). The sensitivity of the creatine kinase assay was 2.2 U·L^−1^, and the intra-assay CV was 0.9%. At the same laboratory visit, body mass and body fat percentage were measured with bioimpedance device (InBody770-analyser; Biospace Co. Ltd., Seoul, Korea).

An incremental treadmill test was performed on a treadmill (Telineyhtymä Oy, Kotka, Finland). The starting speed was 7 km·h^−1^ for women and 8 km·h^−1^ for men. Three-minute stages were used, and the speed increased by 1 km·h^−1^ after every stage. Between the stages, the treadmill was stopped (15–20 s) for drawing blood samples from the fingertip for lactate analyses. The inclination was kept constant at 0.5° angle through the whole test. The oxygen consumption was measured breath by breath with Jaeger Vyntus CPX (CareFusion Germany 234 GmbH, Hoechberg, Germany), and HR was monitored with Polar Vantage V2 (Polar Electro Oy, Kempele, Finland). The maximal oxygen uptake (V̇O_2max_) was defined as the highest 60-s average of oxygen consumption. The maximal running speed (vMax) of the test was defined as the highest speed in the last completed stage, or if the stage was not finished, as the speed of the last completed stage (km·h^−1^) + (running time (s) of the unfinished stage − 30 s)/(180 − 30 s) × 1 km·h^−1^. The first lactate threshold (LT1) and the second lactate threshold (LT2) were determined based on blood lactate changes during the test. The LT1 was set at 0.3 mmol·L^−1^ above the lowest lactate value. For the determination of LT2, two linear models were drawn: 1) between LT1 and the next measured lactate value and 2) for the lactate points, which were preceded by a lactate increase of at least 0.8 mmol·L^−1^. Finally, LT2 was set at the intersection point between these two linear models. The treadmill and threshold assessment protocols were adopted from previous studies (5,14,27).

The countermovement jump (CMJ) test was performed on a contact mat before the incremental treadmill test and after a short 5-min low-intensity warm-up. The participants were advised to keep their hands on their hips and jump as high as possible. The lowest knee angle during the take-off was instructed to be about 90°. The jump height (*h*) was calculated based on the measured flight time with the formula: *h* = *g* · *t*^2^ · 8^−1^, where *t* is the recorded flight time in seconds and *g* is the acceleration due to gravity (9.81 m·s^−2^) (35). Three attempts were performed with a 30-s recovery, and the highest jump (in centimeters) was used in the final analysis.

The running performance was also assessed by the 10-km field test, which was run in small groups on a flat 1.6-km asphalt loop (+400-m starting line). A standardized 15-min low-intensity warm-up including 2–3 accelerations to the target speed was performed before the test. The running time, average HR, and peak HR were analyzed from the tests.

### Training and recovery monitoring

The participants used an HR monitor (Polar Vantage V2, H10 sensor; Polar Electro Oy) in all endurance exercises. The training intensity distribution based on HR values (time below the LT1 = HRzone1; between LT1 and LT2 = HRzone2; above the LT2 = HRzone3), distance covered, HR–RS index (33), and average running speed from the interval sessions were analyzed from the data. To establish a fair comparison between the sessions of varying duration and terrain, the HR–RS index was primarily calculated from the beginning of running sessions (5:00–10:00). The participants were advised to run the first 10 min of each session as a warm-up on flat terrain at an intensity of LIT. The data were manually analyzed in Polar Flow software (Polar Electro Oy) to ensure sufficient data quality and flat terrain requirement (not more than 5 m ascent or descent). In cases where the criteria were not met in the original 5:00–10:00 segment, the 5-min segment was either moved until fulfilling the criteria (continuous sessions), or the longest possible segment (of at least 2 min) meeting the criteria was used (interval sessions) instead.

The HR–RS index was calculated based on the average running speed (*S*_avg_) and HR (HR_avg_) with the following equation:


$$\mathrm{HR}‐\mathrm{RS}\;\text{index}=S_{\mathrm{avg}}-\left({\mathrm{HR}}_{\mathrm{avg}}-{\mathrm{HR}}_{\text{standing}}\right)/k$$

$$k=\left({\mathrm{HR}}_{\max}-{\mathrm{HR}}_{\text{standing}}\right)/S_{\text{peak}}$$

HR_standing_ was estimated by adding 26 bpm to the resting HR (average nocturnal HR during the PREP period) similar to Vesterinen et al. (33). *S*_peak_ and HR_max_ were determined based on the incremental treadmill test results at *T*_1_.

Subjective recovery was estimated daily on a 1–7 scale, which was modified from the questionnaires of Schäfer Olstad et al. (32) and Hooper et al. (30). Muscle soreness of the lower limbs, fatigue, sleep quality, and stress were ranked from 1 (very much below/better than normal) to 7 (very much above/worse than normal), whereas 4 represented normal perception. The items were analyzed separately and as a sum index, which was defined as the “staleness score.” Recovery was estimated in the morning before any exercise via Coach4Pro mobile application (Coach4Pro Oy, Espoo, Finland).

The nocturnal HR and HRV were measured via wrist-based photoplethysmography (Polar Vantage V2) every night throughout the whole study. The validity of the device has been reported previously (36). Automatically formed results from a 4-h period starting half an hour after the beginning of the detected sleep onset were used in the analysis. Values provided by the watch included the average HR and the average root mean square of successive differences, which was log-transformed (LnRMSSD) for the analysis.

### Statistical analysis

The results are presented as mean ± SD. The normality of the data was assessed with the Shapiro–Wilk test. To examine the main effects (time, group) and their interaction (time–group), repeated-measures ANOVA was applied in the performance and laboratory tests (*T*_1_, *T*_2_, *T*_3_), normally distributed monitoring variables (PREP vs 1–12 wk), and the running speed of the interval sessions (INT1 vs INT2–6 wk). In the case of a significant main effect or interaction, a Bonferroni *post hoc* test was used for within-group comparisons and simple contrasts for between-group comparisons. To exclude any possible effects of different baseline levels in performance parameters (treadmill test, 10-km test), a *T*_0_ test result was used as a covariant (ANCOVA) in the between-group analysis. In parameters that were not normally distributed, the Wilcoxon signed rank test with Bonferroni correction was used for within-group comparisons and the Mann–Whitney *U*-test for between-group comparisons (changes from PREP). For the markers used in training adjustment (HR–RS index, 3-d HRV, muscle soreness, and fatigue), unpaired-samples *t*-test was used to analyze the between-group differences in the percentage of data points being within individual SWC during the training intervention. The magnitude of improvements in the main parameters (vMax, 10 km) was analyzed based on the CV between *T*_0_ and *T*_1_ tests, and it was stated as trivial (<0.5 × CV), moderate (0.5–2 × CV), or high (>2 × CV). Because only a relatively short period of regular training was performed between the *T*_0_ and *T*_1_, CV was expected to illustrate the typical error of the test caused by day-to-day variation in performance and/or environmental factors. The present division for magnitude was adapted from the study by Düking et al. (7), but as an exception, the SWC was defined as 0.5 × CV (37), similar to the cutoff value used in the recovery markers. To further investigate possible reasons behind different responses, individuals defined as high responders for both of the tests (*n* = 8) and individuals defined as trivial responders for either of the tests (*n* = 9) were compared (age, baseline fitness, training volume, perceived stress, and recovery during the intervention period) with Mann–Whitney *U*-test. To examine the effect size (ES) of observed changes, Cohen’s *d* for within-group (difference of the means divided by the pooled SD), and between-group (difference of the means divided by the SD of the mean difference) comparisons and 95% confidence intervals were calculated for the laboratory and performance tests. After nonparametric tests, ES was calculated by a formula: ES = *Z* · (√*n*)^−1^, where *Z* is the *z*-score, and *n* is the number of observations on which *Z* is based. The ES was categorized as <0.2 trivial, 0.2–0.5 small, 0.5–0.8 moderate, and >0.8 large. The statistical significance level was set to *P* < 0.05. Analyses were performed with Microsoft Excel 2016 (Microsoft Corporation, Redmond, WA) and IBM SPSS Statistics v.28 programs (SPSS Inc., Chicago, IL).

## RESULTS

### Training

**FIGURE 2:**
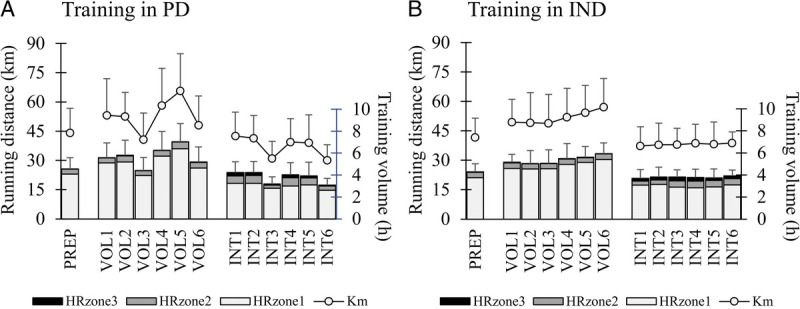
Training volume, running distance, and training intensity distribution (time in HRzone1, HRzone2, and HRzone3) at baseline (PREP) and across the volume (VOL1–VOL6) and interval (INT1–INT6) training periods in the PD (A) and IND (B) training groups.

No differences were observed between the groups in the mean weekly training volume during the PREP (PD, 4.6 ± 1.0 h; IND, 4.3 ± 0.8 h), VOL (PD, 5.7 ± 1.3; IND, 5.3 ± 0.9), or INT (PD, 3.8 ± 0.9; IND, 3.8 ± 0.6 h). Compared with PREP, the training volume was higher during VOL and lower during INT in both groups (*P* < 0.01). The training intensity distribution was similar in both groups across the study. In addition, the proportion of HRzone1 decreased and HRzone3 increased from PREP to INT similarly in both groups (*P* < 0.01). The weekly mean training frequency slightly increased in IND from PREP (4.2 ± 0.6) to VOL (4.4 ± 0.6, *P* < 0.001) and INT (4.3 ± 0.6, *P* = 0.007), whereas no significant differences were found in VOL (PREP, 4.2 ± 0.9; VOL, 4.5 ± 1.0; INT, 4.3 ± 0.9). The number of HIT sessions did not differ between the groups during INT (PD, 13.6 ± 0.5 sessions; IND, 15.8 ± 4.3 sessions), although the range was greater in IND (PD, 13–14 sessions; IND, 10–25 sessions). The weekly training volume and intensity are illustrated in Figure 2. The total accumulated training volume during the VOL and INT was 56.9 ± 13.0 h (range, 43.7–83.9 h) in PD and 54.7 ± 9.0 h (range, 40.3–69.1 h) in IND, and the volume was distributed into 52 ± 11 sessions (range, 42–80 sessions) in PD and 53 ± 7 sessions (range, 46–71 sessions) in IND. Regarding the training adjustments of IND during the intervention, 55% ± 12% maintained the training load, 35% ± 10% increased the training load, and 10% ± 8% decreased the training load.

### Performance and laboratory tests

No between-group differences were observed in any of the performance-related variables at *T*_1._ A significant main effect of time was observed in vLT2, vMax, and V̇O_2max_ (*P* < 0.001; Table 3). Both groups improved (*P* < 0.001) their maximal treadmill performance from *T*_1_ to *T*_3_ (PD, 3.0% ± 2.4%; IND, 4.0% ± 1.9%; between-group *P* = 0.322; ES = 0.46; −0.27 to 1.18), and *T*_2_ to *T*_3_ (PD, 1.8% ± 2.5% (*P* = 0.022); IND, 2.7% ± 2.8% (*P* = 0.001); between-group *P* = 0.421; ES = 0.34; −0.39 to 1.06). No significant main effects or interactions were observed in the anthropometrics or blood-derived markers (Table 3).

**TABLE 3:** Mean ± SD performance and laboratory test results before the VOL (*T*_1_) between the VOL and INT (*T*_2_), and after the INT (*T*_3_) periods.

	PD (*n* = 14)			IND (*n* = 16)		
	*T*_1_	*T*_2_	*T*_3_	*T*_1_	*T*_2_	*T*_3_
vLT1 (km·h^−1^)	10.7 ± 0.9	10.8 ± 1.1 (0.10; −0.43 to 0.63)	11.1 ± 1.2 (0.33; −0.21 to 0.87)	10.6 ± 1.1	10.8 ± 1.0 (0.23; −0.27 to 0.72)	10.8 ± 1.3 (0.14; −0.36 to 0.63)
vLT2 (km·h^−1^)	13.3 ± 1.4	13.5 ± 1.5 (0.15,. −38 to 0.67)	13.6 ± 1.6* (0.18; −0.35 to 0.70)	13.1 ± 1.6	13.3 ± 1.4 (0.16; −0.33 to 0.65)	13.5 ± 1.6** (0.23; −0.27 to 0.72)
vMax (km·h^−1^)	16.1 ± 1.8	16.3 ± 2.0 (0.11; −0.42 to 0.63)	16.6 ± 2.1***^,†^ (0.26; −0.28 to 0.79)	16.0 ± 2.0	16.2 ± 2.0 (0.10; −0.39 to 0.59)	16.6 ± 1.9***^,††^ (0.32; −0.19 to 0.82)
V̇O_2max_ (mL·kg^−1^·min^−1^)	46.7 ± 3.9	47.8 ± 5.2 (0.26; −0.28 to 0.78)	50.7 ± 6.1***^,†††^ (0.80; −0.18;1.39)	47.3 ± 7.2	47.0 ± 7.2 (−0.03; −0.49 to 0.44)	50.3 ± 7.6**^,†††^ (0.40; −0.12 to 0.90)
CMJ (cm)	28.0 ± 5.2	28.8 ± 4.7 (0.15; −0.38 to 0.67)	28.6 ± 4.4 (0.11; −0.41 to 0.64)	30.3 ± 6.3	30.6 ± 6.8 (0.05; −0.44 to 0.54)	30.0 ± 6.2 (−0.05; −0.54 to 0.44)
fTesto (pmol·L^−1^)	27.0 ± 26.6	26.9 ± 25.5 (0.00; −0.69 to 0.69)	27.3 ± 23.3 (0.00; −0.69 to 0.69)	18.6 ± 17.5	18.2 ± 16.8 (−0.01; −0.75 to 0.73)	18.6 ± 16.4 (0.00; −0.74 to 0.74)
Cortisol (nmol·L^−1^)	382 ± 102	439 ± 107 (0.50; −0.07;1.04)	411 ± 96 (0.24; −0.30 to 0.76)	464 ± 145	468 ± 145 (0.02; −0.47 to 0.51)	456 ± 125 (−0.06; −0.55 to 0.43)
CK (umol·L^−1^)	130 ± 63	148 ± 90 (0.06; −0.63 to 0.75)	115 ± 54 (−0.07; −0.76 to 0.62)	118 ± 55	130 ± 65 (0.05; −0.69 to 0.79)	129 ± 79 (0.05; −0.69 to 0.79)

Values in the parentheses are ES (Cohen’s *d*) and the 95% confidence intervals for the within-group changes from *T*_1_.

*P < 0.05 within groups compared with *T*_1_.

**P < 0.01 within groups compared with *T*_1_.

***P < 0.001 within groups compared with *T*_1_.

†*P* < 0.05 within groups compared with *T*_2_.

††*P* < 0.01 within groups compared with *T*_2_.

†††*P* < 0.001 within groups compared with *T*_2_.

CK, serum creatine kinase. fTesto, serum free testosterone; vLT1, the speed at the first lactate threshold; vLT2, the speed at the second lactate threshold; vMax, maximal speed of the incremental treadmill test.

**FIGURE 3:**
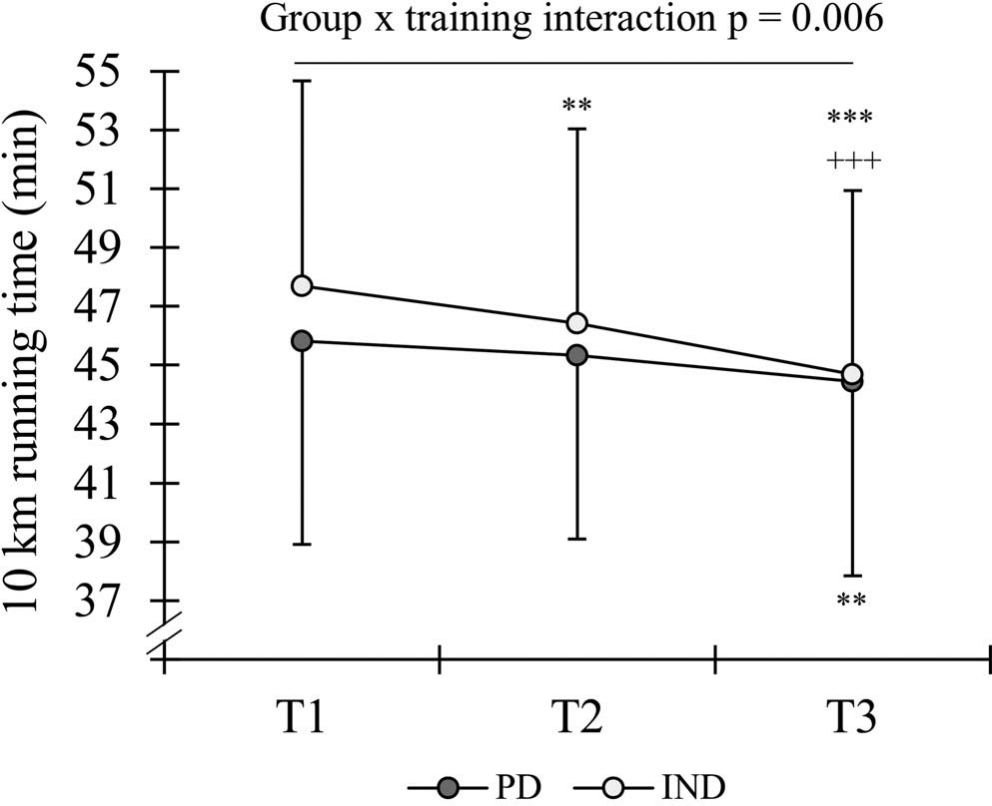
Running time in the 10-km test before the VOL (*T*_1_), between the VOL and INT (*T*_2_), and after the INT (*T*_3_) periods in the PD and IND training groups. **P < 0.01, ***P < 0.001 within groups compared with *T*_1_ +++*P* < 0.001 within groups compared with *T*_2_.

A significant main effect of time (*P* < 0.001) and group–time interaction (*P* = 0.006) was observed in 10-km running time (Fig. 3). PD (−2.9% ± 2.4%, *P* = 0.004; ES = 0.20; −0.35 to 0.75) and IND (−6.2% ± 2.8%, *P* < 0.001; ES = 0.46; −0.07 to 0.97) improved the 10-km running time from *T*_1_ to *T*_3_, and the respective change differed between the groups (*P* = 0.002; ES = 1.23; 0.42 to 2.02). The running time was improved from *T*_1_ to *T*_2_ only in IND (−2.6% ± 3.1%, *P* = 0.001; ES = 0.19; −0.31 to 0.68), whereas in PD, it remained unchanged (−0.8% ± 2.1%, *P* = 0.534; ES = 0.08; −0.47 to 0.62). However, the change was not different between groups (*P* = 0.125; ES = 0.64; −0.12 to 1.38). The improvement was also significant between *T*_2_ and *T*_3_ in IND (−3.7 ± 2.2, *P* < 0.001; ES = 0.27; −0.23 to 0.76) and tended to be significant in PD (−2.0% ± 3.3%, *P* = 0.051; ES = 0.14; −0.41 to 0.68) with no between-group differences (*P* = 0.087; ES = 0.61; −0.15 to 1.35).

Significant main effects of time were also observed in average HR (*P* = 0.035) and peak HR (*P* = 0.002) during the running test. The average HR values at *T*_1_, *T*_2_, and *T*_3_ were 93.1 ± 2.1, 93.3 ± 1.6, and 92.6 ± 2.5%/max for PD and 93.1 ± 1.6, 93.4 ± 1.9, and 92.5 ± 2.1%/max for IND, respectively. At the same time points, peak HR values were 99.0 ± 2.3, 98.5 ± 1.6, and 97.7 ± 1.9%/max for PD and 99.2 ± 2.0, 99.0 ± 2.0, and 97.5 ± 2.2%/max for IND, respectively. In the *post hoc* analysis, the only significant difference was found in peak HR, which decreased in IND from *T*_1_ to *T*_3_ (*P* = 0.011).

**FIGURE 4:**
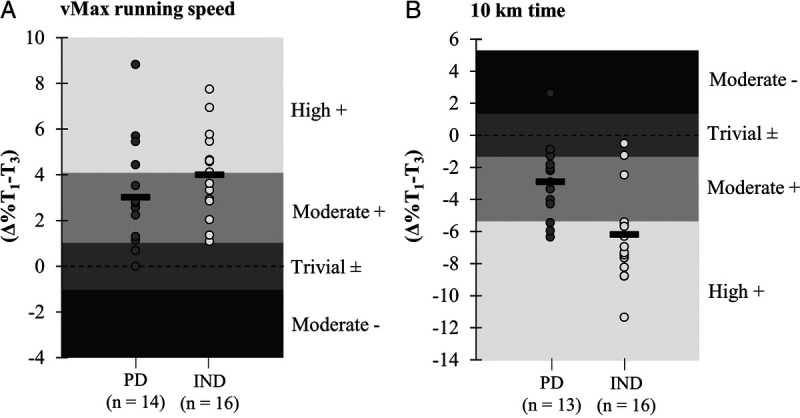
Magnitude of individual responses and mean changes (*black rectangle*) in maximal treadmill speed (A) and 10-km running time (B). Magnitudes were set based on the CV of the parameter between *T*_0_ and *T*_1_. High + and Moderate + indicate improved performance; Trivial ±, unchanged performance; and Moderate −, impaired performance.

In addition to statistical analysis, the individual response magnitudes in the maximal treadmill performance and 10-km running performance from *T*_1_ to *T*_3_ were examined (Fig. 4). In the vMax, the percentage distributions for high, moderate, and trivial responders were 29%/50%/21% for PD and 50%/50%/0% for IND, respectively. Meanwhile, in the 10-km running test, the percentage distributions for high, moderate, trivial, and moderate negative responders were 23%/54%/15%/8% for PD and 81%/6%/13%/0% for IND, respectively.

### Monitoring variables

**FIGURE 5:**
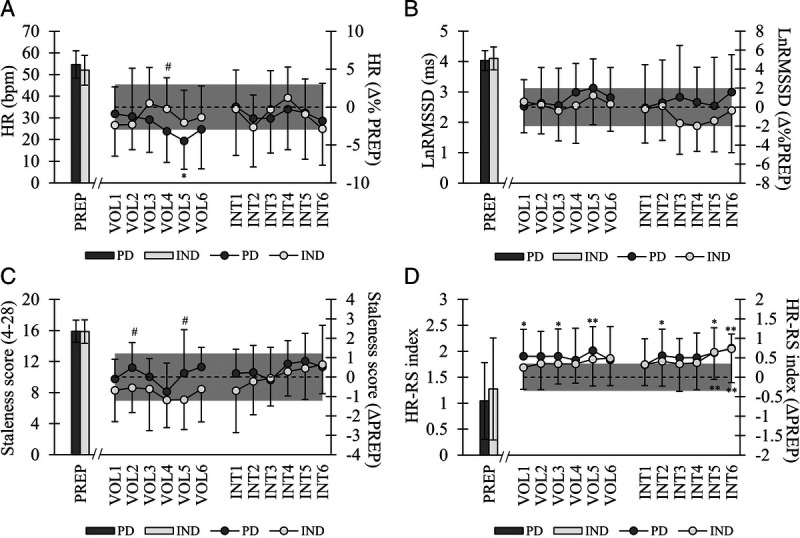
Mean ± SD baseline values (PREP) and weekly changes (VOL1–6, INT1–6) in nocturnal HR (A), nocturnal LnRMSSD (B), staleness score (C), and HR–RS index (D). *Gray area* represents the SWC of the parameter based on individual average values during PREP. In A and B = 0.5 × CV, in C and D = 0.5 × SD. *P < 0.05, **P < 0.01, ***P < 0.001 within groups compared with PREP. #*P* < 0.05 between groups at respective time point.

Significant main effects of time were observed in the HR–RS index (Fig. 5) and the average running speed of interval sessions (*P* < 0.001). The running speed in the intervals increased in IND from week 1 (14.4 ± 1.6 km·h^−1^) to week 3 (14.8 ± 1.8 km·h^−1^, *P* = 0.023), week 4 (14.8 ± 1.8 km·h^−1^, *P* = 0.005), and week 6 (14.9 ± 1.8 km·h^−1^, *P* = 0.023), whereas no change was observed in PD (14.6 ± 2.0 vs 14.7–14.9 km·h^−1^). In addition, some significant within-group differences were found in the staleness score and nocturnal HR (Fig. 5), which were analyzed with nonparametric tests. IND had significantly higher proportion defined as “normal” in HR–RS index (82% ± 6% vs 75% ± 7%, *P* = 0.015) and LnRMSSD (52% ± 5% vs 45% ± 5%, *P* = 0.046) when the percentage of data points being within individual SWC was analyzed, whereas in fatigue (68% ± 11% vs 75% ± 14%) and muscle soreness (69% ± 17% vs 69% ± 24%), no differences were observed.

### Comparison between high and trivial responders

When the individuals defined as high or trivial responders were compared, no differences were observed in the age (34.3 ± 8.6 vs 36.0 ± 6.5 yr), baseline fitness (vMax, 15.5 ± 1.9 vs 15.9 ± 2.0 km·h^−1^), training volume (56.1 ± 6.5 vs 56.6 ± 13.3 h), or perceived stress (3.5 ± 0.5 vs 3.8 ± 0.6) during the study period. Regarding the monitoring variables, high responders had a higher proportion (*P* = 0.03) of “normal” HR–RS index values compared with trivial responders (82% ± 6% vs 73% ± 8%), whereas in LnRMSSD (53% ± 7% vs 49% ± 14%), fatigue (76% ± 16% vs 72% ± 12%), or muscle soreness (76% ± 15% vs 70% ± 16%), no such differences were observed.

## DISCUSSION

The main findings of the study were that the predefined and individualized training protocols improved endurance performance from the baseline in the incremental treadmill test and 10-km running test, and the most significant improvements occurred after the interval period. Although both groups had similar training characteristics on average, the change in the 10-km running performance was greater in IND. In addition, the proportion of high responders in the maximal treadmill and 10-km running performance was greater and the proportion of low responders smaller in IND compared with PD. These differences suggest that individualized training may increase the likelihood of positive endurance training adaptations.

### Training characteristics

Despite different training periodization models, no significant differences were found between the groups when training periods were analyzed as a whole. However, as can be seen from Figure 2, the weekly execution of the training was quite different. In IND, similar types of recovery weeks as in PD were not observed, because the timing and length of such periods were individually defined. Interestingly, only ~10% of the training load adjustments led to a recovery block. This may illustrate that in recreational runners with quite a low training frequency, specific recovery periods may not be particularly critical when the training load is being increased (sufficiently) moderately. The findings may also relate to somewhat strict limits for the recovery block, at least in some individuals.

Previously, HRV-guided training has led not only to a lower volume of MOD or HIT (13,14,16) but also to a higher volume of MOD (17). In the present study, there was rather a slight tendency for a higher proportion of HIT during INT (IND vs PD, 15.6 vs 13.6 sessions), but the difference was not significant because there were also many individuals who performed fewer HIT sessions than PD. Although it has been previously found that the same (or superior) adaptations could be induced with lower training demands of HRV-guided training (13,14,16), one may argue that the training characteristics should be, on average, similar between the two groups to indicate that also the PD group has a suitable program.

### Training adaptation

Both groups improved significantly their maximal performance in the incremental treadmill test and 10-km running test. The magnitudes of improvements in the vMax (PD, 3.0% ± 2.4%; IND, 4.0% ± 1.9%) (5,21) and 10-km running tests (PD, −2.9% ± 2.4%; IND, −6.2% ± 2.8%) (38,39) were in line with the aforementioned previous studies, which suggests that the training programs were appropriate for the target population of recreational runners. The most interesting finding among the performance tests was the significant difference between PD and IND in the change of 10-km running time. Regarding the greater between-group difference in 10 km compared with the treadmill test, one possible explanation could be related to the timing of the test. Because IND did not have a predefined recovery or tapering period before the test week, it is possible that, during the latter test day of the week, (10 km), the training adaptations and the actual performance were better realized. Although maximal treadmill and 10-km running performance are strongly linked (40), the 10-km test may provide information from slightly different aspects of endurance performance by requiring “durability” of high intensity for a prolonged period (41), which is quite a critical ability in most endurance events. It can also be speculated that maximal treadmill performance could be limited by neuromuscular factors (42), especially in recreational runners, since the 10-km running speed was, on average, 82% of the peak treadmill speed.

The greater number of high responders and the lower number of trivial or negative responders in the IND group were another interesting findings regarding the training adaptations in vMax and 10 km. This is in line with the hypothesis that individualizing the training load would decrease the likelihood of negative responses. Similar findings have also been proposed by Vesterinen et al. (14), who suggested that HRV-guided training would decrease the variation in the training adaptation and lead to more consistent improvements in performance. On the other hand, the lack of changes in the treadmill performance after the volume period was rather an unexpected result. For optimal performance after VOL, the current protocol would possibly have required longer tapering or a greater decrease in volume before the tests. In the study by Bellinger et al. (25), using quite a similar volume progression, the running performance was significantly improved after a 1-wk taper during which the training volume was exponentially decreased by 55%.

### Monitoring variables

In the monitoring variables, only a few differences were observed between the groups, and none of the markers responded negatively at the group level. Furthermore, the resting concentrations of serum hormones and CMJ performance remained unaffected. Therefore, the training load seemed tolerable for both groups. Regarding the between-group differences in the monitoring variables, IND had a higher proportion of “normal” values in HR–RS index and nocturnal HRV, which was an expected outcome of the training model. Although both groups improved the HR–RS index, only IND was able to increase the running speed significantly during the interval sessions. Because maximal sustainable effort intervals could be regarded as a marker of the current performance level (27), the finding may illustrate a compromised training state in some individuals of PD, probably because of too high interval frequency. The importance of maintaining an appropriate training state was also demonstrated by a greater proportion of “normal” values in HR–RS index in high responders compared with trivial responders.

Although a positive state of recovery is in general desirable, at least a slight variation in these markers might be necessary at certain points of training periods to reflect a sufficient training load needed for long-term improvements. It is important to acknowledge that individualized training may allow not only sufficient recovery but also sufficient loading to induce desirable adaptations. This could also relate to the lesser occurrence of low responders in the current study. Montero and Lundby (43) have previously found that individuals stated as nonresponders improved their endurance performance when the training dose was increased. Gaskill et al. (44) illustrated the same phenomenon from a different perspective, and in their study, the individuals who were stated as low-responders to previous training improved their performance once the training was significantly intensified.

### Methodological considerations

The current study setting was novel, and no previous recommendations exist regarding the multitargeted training model of the IND group. Therefore, several considerations based on the observations made in the present study may be beneficial for future studies or individuals implementing such an approach into practice.

First, the markers used in the recovery evaluation play a critical role, and therefore, the selection of proper markers should be considered carefully. HRV was chosen as an evaluation marker based on previous studies utilizing individualized training prescription. In all previous studies using the HRV-guided approach, morning recordings (13–17) or day-time recordings (12) have been used instead of nocturnal recordings. In the present study, nocturnal recordings were chosen because of feasibility, as they did not demand any additional measurements. Although sleep is not necessarily a stable period in terms of the autonomic nervous system function and HRV (45) when data are being averaged for a sufficient period (e.g., 4 h), a very good day-to-day reliability has been reported (46) and within-week variation could be even lower compared with morning recordings (47). Furthermore, nocturnal HRV seems to be sensitive and demonstrate internal responses to training load (46). Subjective markers are typically suggested to be useful tools in the detection of overreaching or overtraining (30,31) and helpful in distinguishing positive and negative responses in HR-based markers, such as resting HRV and submaximal exercise HR (22,23). Fatigue and muscle soreness were used in the present study, because both of these have previously been associated with staleness (30) and responded to a significant increment in the training load (23). The most useful subjective markers would probably be those that provide information from a point of view that could not be assessed via objective measures, and simple assessments consisting of only a few aspects of subjective recovery could be the most suitable and practical option (32).

In addition to the recovery state, the estimations of performance provide information on the training adaptation, which is the ultimate goal of the whole training process. In the current study, the submaximal performance was assessed at the beginning of each exercise via the HR–RS index. Previously, similar types of warm-up settings (5–10 min) have been able to capture acute (4) and chronic (38) changes in HR in the standardized conditions. Despite the fact that the current setting was not similarly standardized by treadmill or beep sounds, the use of the HR–RS index was expected to equalize slight variations in speed or HR. In addition, Vesterinen et al. (48) have found that a 15-min HR-based warm-up in field conditions was able to track training adaptation in the laboratory test. Although submaximal performance correlates with the maximal performance with a decent accuracy, especially HR-based tests have certain challenges in terms of interpretation. In the present study, it was expected that if submaximal HR decreased because of overreaching, this type of parasympathetic hyperactivity would be revealed via increased perceived fatigue and HRV (22,23). Another option to exclude HR-based challenges would be testing running speed in relation to fixed rating of perceived exertion (49), for example, with a similar warm-up setting compared with the present study.

Another important aspect to consider when assessing recovery is the limits/normal range within each variable. Although, in subjective markers, the desirable values may remain quite permanent, in the HR-based and performance-related markers, there is an occasional need for reevaluation, because these may change because of positive training adaptations (38). The frequency of such evaluations has varied in previous studies from constant updating (12,13) to updating once per week (17) or once every 4 wk (14,16). In the current study, constantly updated limits were used, and some individuals illustrated slow downward slipping of the limits (e.g., in HR–RS index), which would not be desirable. Therefore, if using constantly updating SWC in particular, one way to avoid such an effect would be to set a short-term limit (e.g., 2-wk) and a long-term limit (e.g., 8-wk), and both of them should be met. Regarding the exact cutoff values for each variable, it is possible to set a desirable “risk level,” which could vary, for example, depending on the training phase or fitness level.

The final step of the individualization consists of the following question: how training should be adjusted based on the results? In previous studies, individualized training prescriptions have been utilized purely via adjustment of intensity (12–17,24). The training volume, however, is a critical variable in the long-term development of endurance performance (19,50), and consequently, a model that only estimates whether an individual should train at high- or low-intensity could be regarded as somewhat incomplete. Therefore, we argue that also the training volume should be considered in the training decision scheme. Regarding the training execution, previous HRV-guided studies have mainly utilized “day-by-day-approach” (13,14,16,17). Based on the results of the current study, a 3- to 4-day evaluation period seemed a relevant option in terms of feasibility (individuals know the session of the following day) and training load that would not lead to a serious state of fatigue or overreaching, where the recovery period would be extended (1). Nevertheless, it should be noted that, in the present study the average training frequency was only slightly greater than four sessions per week, thus allowing always fairly decent recovery periods between sessions in most of the participants.

Finally, although the idea behind individualized training is that the training is adjusted based on data collected, in the long term, predefined recovery periods (e.g., every fifth week) may secure exclusion of excessive fatigue. It could also be beneficial for the perceptual aspects of recovery, which are likely to get impaired during intensive training (22,23,27). Even if the training was adapted, there probably should always be upper and lower limits for the acute and long-term progression of the training load, and these should be determined based on the individual’s background and target.

### Limitations

In the current study, males and females were analyzed within the same group, because the number of participants did not allow meaningful separate comparisons. Further studies are needed to investigate possible sex differences and to elaborate current findings to cover untrained and competitive athletes, although it can be argued that a similar necessity for the balance between training load and recovery exists across the fitness-level spectrum. The study was performed in “field conditions”; thus, training conditions or factors such as nutrition or hydration status could not be fully controlled. In addition, the 10-km running test was performed outdoors where environmental factors could not be standardized at similar precision as in the laboratory. However, similar fluctuations in the conditions concerned both groups, and therefore, environmental factors most likely did not affect significantly within-group comparisons. It could also be argued that the current field setting reflects the conditions of the “real” training of recreational runners and thus the usefulness of both training models.

## CONCLUSIONS

In conclusion, the current study provided evidence that, although predefined training improves endurance performance, individualized endurance training may induce greater improvements in running performance and increase the probability of high response while decreasing the occurrence of low or negative responses to endurance training. In the future, the most suitable markers to be used in monitoring as well as the exact method of how training load could be manipulated during different types of periods should be examined in more detail.

## References

[1] Meeusen R Duclos M Foster C, European College of Sport Science; American College of Sports Medicine. Prevention, diagnosis, and treatment of the overtraining syndrome: joint consensus statement of the European College of Sport Science and the American College of Sports Medicine. *Med Sci Sports Exerc*. 2013;45(1):186–205. 23247672 10.1249/MSS.0b013e318279a10a

[2] Seiler S Haugen O Kuffel E. Autonomic recovery after exercise in trained athletes: intensity and duration effects. *Med Sci Sports Exerc*. 2007;39(8):1366–73. 17762370 10.1249/mss.0b013e318060f17d

[3] Flatt AA Globensky L Bass E Sapp BL Riemann BL. Heart rate variability, neuromuscular and perceptual recovery following resistance training. *Sports (Basel)*. 2019;7(10):225. 10.3390/sports7100225 PMC6835520 31635206

[4] Nuuttila OP Kyröläinen H Häkkinen K Nummela A. Acute physiological responses to four running sessions performed at different intensity zones. *Int J Sports Med*. 2021;42(6):513–22. 33176386 10.1055/a-1263-1034

[5] Vesterinen V Häkkinen K Laine T Hynynen E Mikkola J Nummela A. Predictors of individual adaptation to high-volume or high-intensity endurance training in recreational endurance runners. *Scand J Med Sci Sports*. 2016;26(8):885–93. 26247789 10.1111/sms.12530

[6] Zinner C Schäfer Olstad D Sperlich B. Mesocycles with different training intensity distribution in recreational runners. *Med Sci Sports Exerc*. 2018;50(8):1641–8. 29509644 10.1249/MSS.0000000000001599

[7] Düking P Holmberg HC Kunz P Leppich R Sperlich B. Intra-individual physiological response of recreational runners to different training mesocycles: a randomized cross-over study. *Eur J Appl Physiol*. 2020;120(12):2705–13. 32918588 10.1007/s00421-020-04477-4 PMC7674349

[8] Stellingwerff T Heikura IA Meeusen R, . Overtraining syndrome (OTS) and relative energy deficiency in sport (RED-S): shared pathways, symptoms and complexities. *Sports Med*. 2021;51(11):2251–80. 34181189 10.1007/s40279-021-01491-0

[9] Halson SL. Nutrition, sleep and recovery. *Eur J Sport Sci*. 2008;8(2):119–26.

[10] Ruuska PS Hautala AJ Kiviniemi AM Mäkikallio TH Tulppo MP. Self-rated mental stress and exercise training response in healthy subjects. *Front Physiol*. 2012;3:51. 22416235 10.3389/fphys.2012.00051 PMC3298959

[11] Halson SL. Monitoring training load to understand fatigue in athletes. *Sports Med*. 2014;44(2 Suppl):S139–47. 25200666 10.1007/s40279-014-0253-z PMC4213373

[12] da Silva DF Ferraro ZM Adamo KB Machado FA. Endurance running training individually guided by HRV in untrained women. *J Strength Cond Res*. 2019;33(3):736–46. 28570494 10.1519/JSC.0000000000002001

[13] Kiviniemi AM Hautala AJ Kinnunen H Tulppo MP. Endurance training guided individually by daily heart rate variability measurements. *Eur J Appl Physiol*. 2007;101(6):743–51. 17849143 10.1007/s00421-007-0552-2

[14] Vesterinen V Nummela A Heikura I, . Individual endurance training prescription with heart rate variability. *Med Sci Sports Exerc*. 2016;48(7):1347–54. 26909534 10.1249/MSS.0000000000000910

[15] Nuuttila OP Nikander A Polomoshnov D Laukkanen JA Häkkinen K. Effects of HRV-guided vs. predetermined block training on performance, HRV and serum hormones. *Int J Sports Med*. 2017;38(12):909–20. 28950399 10.1055/s-0043-115122

[16] Javaloyes A Sarabia JM Lamberts RP Moya-Ramon M. Training prescription guided by heart rate variability in cycling. *Int J Sports Physiol Perform*. 2019;14(1):23–32. 10.1123/ijspp.2018-0122 29809080

[17] Carrasco-Poyatos M González-Quílez A Altini M Granero-Gallegos A. Heart rate variability-guided training in professional runners: effects on performance and vagal modulation. *Physiol Behav*. 2022;244:113654. 34813821 10.1016/j.physbeh.2021.113654

[18] Stanley J Peake JM Buchheit M. Cardiac parasympathetic reactivation following exercise: implications for training prescription. *Sports Med*. 2013;43(12):1259–77. 23912805 10.1007/s40279-013-0083-4

[19] Laursen PB. Training for intense exercise performance: high-intensity or high-volume training? *Scand J Med Sci Sports*. 2010;20(2 Suppl):1–10. 10.1111/j.1600-0838.2010.01184.x 20840557

[20] Thamm A Freitag N Figueiredo P, . Can heart rate variability determine recovery following distinct strength loadings? A randomized cross-over trial. *Int J Environ Res Public Health*. 2019;16(22):4353. 10.3390/ijerph16224353 PMC6888606 31703468

[21] Nuuttila OP Nummela A Häkkinen K Seipäjärvi S Kyröläinen H. Monitoring training and recovery during a period of increased intensity or volume in recreational endurance athletes. *Int J Environ Res Public Health*. 2021;18(5):2401. 33804541 10.3390/ijerph18052401 PMC7967764

[22] Le Meur Y Pichon A Schaal K, . Evidence of parasympathetic hyperactivity in functionally overreached athletes. *Med Sci Sports Exerc*. 2013;45(11):2061–71. 24136138 10.1249/MSS.0b013e3182980125

[23] Bellenger CR Karavirta L Thomson RL Robertson EY Davison K Buckley JD. Contextualizing parasympathetic hyperactivity in functionally overreached athletes with perceptions of training tolerance. *Int J Sports Physiol Perform*. 2016;11(7):685–92. 26640275 10.1123/ijspp.2015-0495

[24] Capostagno B Lambert MI Lamberts RP. Standardized versus customized high-intensity training: effects on cycling performance. *Int J Sports Physiol Perform*. 2014;9(2):292–301. 23881116 10.1123/ijspp.2012-0389

[25] Bellinger P Desbrow B Derave W, . Muscle fiber typology is associated with the incidence of overreaching in response to overload training. *J Appl Physiol (1985)*. 2020;129(4):823–36. 32816636 10.1152/japplphysiol.00314.2020

[26] Bosquet L Montpetit J Arvisais D Mujika I. Effects of tapering on performance: a meta-analysis. *Med Sci Sports Exerc*. 2007;39(8):1358–65. 17762369 10.1249/mss.0b013e31806010e0

[27] Nuuttila OP Nummela A Kyröläinen H Laukkanen J Häkkinen K. Physiological, perceptual, and performance responses to the 2-wk block of high- versus low-intensity endurance training. *Med Sci Sports Exerc*. 2022;54(5):851–60. 35072660 10.1249/MSS.0000000000002861 PMC9012527

[28] Helgerud J Høydal K Wang E, . Aerobic high-intensity intervals improve VO_2_max more than moderate training. *Med Sci Sports Exerc*. 2007;39(4):665–71. 17414804 10.1249/mss.0b013e3180304570

[29] Seiler S Jøranson K Olesen BV Hetlelid KJ. Adaptations to aerobic interval training: interactive effects of exercise intensity and total work duration. *Scand J Med Sci Sports*. 2013;23(1):74–83. 21812820 10.1111/j.1600-0838.2011.01351.x

[30] Hooper SL Mackinnon LT Howard A Gordon RD Bachmann AW. Markers for monitoring overtraining and recovery. *Med Sci Sports Exerc*. 1995;27(1):106–12. 7898325

[31] Ten Haaf T van Staveren S Oudenhoven E, . Prediction of functional overreaching from subjective fatigue and readiness to train after only 3 days of cycling. *Int J Sports Physiol Perform*. 2017;12(2 Suppl):S287–94. 27834554 10.1123/ijspp.2016-0404

[32] Schäfer Olstad DS Borgen-Johansen M Garthe I Paulsen G. Responsiveness of time and cost-efficient monitoring measures on triathlon training a call for individualized training monitoring. *J Athl Enhanc*. 2019;8(5):1–6.

[33] Vesterinen V Hokka L Hynynen E Mikkola J Häkkinen K Nummela A. Heart rate-running speed index may be an efficient method of monitoring endurance training adaptation. *J Strength Cond Res*. 2014;28(4):902–8. 24345970 10.1519/JSC.0000000000000349

[34] Lamberts RP Lambert MI. Day-to-day variation in heart rate at different levels of submaximal exertion: implications for monitoring training. *J Strength Cond Res*. 2009;23(3):1005–10. 19387374 10.1519/JSC.0b013e3181a2dcdc

[35] Bosco C Luhtanen P Komi PV. A simple method for measurement of mechanical power in jumping. *Eur J Appl Physiol Occup Physiol*. 1983;50(2):273–82. 6681758 10.1007/BF00422166

[36] Nuuttila OP Korhonen E Laukkanen J Kyröläinen H. Validity of the wrist-worn Polar Vantage V2 to measure heart rate and heart rate variability at rest. *Sensors (Basel)*. 2021;22(1):137. 35009680 10.3390/s22010137 PMC8747571

[37] Hopkins WG. Competitive performance of elite track-and-field athletes: variability and smallest worthwhile enhancements. *Sportscience*. 2005;9:17–20.

[38] Buchheit M Chivot A Parouty J, . Monitoring endurance running performance using cardiac parasympathetic function. *Eur J Appl Physiol*. 2010;108(6):1153–67. 20033207 10.1007/s00421-009-1317-x

[39] Muñoz I Seiler S Bautista J España J Larumbe E Esteve-Lanao J. Does polarized training improve performance in recreational runners? *Int J Sports Physiol Perform*. 2014;9(2):265–72. 23752040 10.1123/ijspp.2012-0350

[40] Noakes TD Myburgh KH Schall R. Peak treadmill running velocity during the VO_2_ max test predicts running performance. *J Sports Sci*. 1990;8(1):35–45. 2359150 10.1080/02640419008732129

[41] Maunder E Seiler S Mildenhall MJ Kilding AE Plews DJ. The importance of ‘durability’ in the physiological profiling of endurance athletes. *Sports Med*. 2021;51(8):1619–28. 33886100 10.1007/s40279-021-01459-0

[42] Paavolainen L Nummela A Rusko H. Muscle power factors and VO_2_max as determinants of horizontal and uphill running performance. *Scand J Med Sci Sports*. 2000;10(5):286–91. 11001396 10.1034/j.1600-0838.2000.010005286.x

[43] Montero D Lundby C. Refuting the myth of non-response to exercise training: ‘non-responders’ do respond to higher dose of training. *J Physiol*. 2017;595(11):3377–87. 28133739 10.1113/JP273480 PMC5451738

[44] Gaskill SE Serfass RC Bacharach DW Kelly JM. Responses to training in cross-country skiers. *Med Sci Sports Exerc*. 1999;31(8):1211–7. 10449026 10.1097/00005768-199908000-00020

[45] Herzig D Eser P Omlin X Riener R Wilhelm M Achermann P. Reproducibility of heart rate variability is parameter and sleep stage dependent. *Front Physiol*. 2018;8:1100. 29367845 10.3389/fphys.2017.01100 PMC5767731

[46] Nummela A Hynynen E Vesterinen V. Nocturnal heart rate and heart rate variability as a method for monitoring training load. In: Kokusuz F Ertan H Tsolakidis E, ed. 15th Annual Congress of the European College of Sport Science; 2010 Jun 23–26: Antalya (Turkey): Book of Abstracts; 2010, p. 516.

[47] Mishica C Kyröläinen H Hynynen E Nummela A Holmberg HC Linnamo V. Evaluation of nocturnal vs. morning measures of heart rate indices in young athletes. *PLoS One*. 2022;17(1):e0262333. 34986202 10.1371/journal.pone.0262333 PMC8730395

[48] Vesterinen V Nummela A Äyramo S, . Monitoring training adaptation with a submaximal running test under field conditions. *Int J Sports Physiol Perform*. 2016;11(3):393–9. 26308590 10.1123/ijspp.2015-0366

[49] Sangan HF Hopker JG Davison G McLaren SJ. The self-paced submaximal run test: associations with the graded exercise test and reliability. *Int J Sports Physiol Perform*. 2021;16(12):1865–73. 34140417 10.1123/ijspp.2020-0904

[50] Seiler S. What is best practice for training intensity and duration distribution in endurance athletes? *Int J Sports Physiol Perform*. 2010;5(3):276–91. 20861519 10.1123/ijspp.5.3.276

